# Dewatering and valorizing lake sediments by electroosmotic dewatering for lakes restoration

**DOI:** 10.1007/s11356-024-33935-1

**Published:** 2024-06-14

**Authors:** Huilin Li, Lisbeth M. Ottosen

**Affiliations:** Department of Environmental and Resource Engineering, Technical University of Denmark, Lyngby, Denmark

**Keywords:** Electrochemical treatment, Electroosmosis, Lake sediments, Resource recovery, Moisture distribution, Dewaterability

## Abstract

Dredging eutrophic lake sediments can improve water quality, but it also requires dewatering and valorizing the dredged material to avoid wasting resources like phosphorus. This study experimentally investigated the basic mechanism and performance of electroosmotic dewatering of 1-L dredged sediments using different electric currents (20 mA, 40 mA, and 60 mA) after gravity filtration. The dewatering performance, moisture content and distribution, effect of electrochemical reaction on dewaterability, energy consumption, and changes in metals and phosphorus (P) distribution and pH values were analyzed. The results indicated that electroosmotic dewatering effectively decreased sediment mass by predominantly eliminating free and a portion of interstitial water, with reductions ranging from 7 to 20%. The optimal duration and current should, however, be considered to balance water removal and energy consumption. Higher moisture removal occurred with 40 mA for 24 h and 60 mA for 6 h, while the energy consumption obtained with 60 mA (0.201 kWh/kg _water removed_) was significantly lower than that of applying 40 mA for 24 h (0.473 kWh/kg _water removed_), with the assistance of ohmic heating, resulting in reduced viscosity and water release from capillaries. The tested conditions did not significantly extract heavy metals or P from the sediments, which may facilitate the disposal of the removed water back into the lake and the utilization of the treated sediments for different purposes. This technology is easy to operate and suitable for the treatment of dredged sediments, and the dewatering result is comparable to low pressurized filtration but at low energy consumption.

## Introduction

Many lakes suffer from eutrophication (Ikem and Adisa 2011; Zhang et al. 2020). Dredging the sediment is one way to deal with this problem, as it enhances the water quality and preserves the waterway’s function (Zhang et al. 2016). However, dredging produces a large amount of sediment that needs to be disposed of. If these sediments are disposed of in a landfill, valuable resources within the sediment are wasted. One of the resources is phosphorous (P), which is listed in the EU list of critical resources (Ottosen et al. 2013, 2016; Villen-Guzman et al. 2018; European Commission 2023).

Dredged sediments have a very high-water content and dewatering is necessary to make them easier to transport and store. Some common mechanical dewatering methods are gravity settlers, centrifuges, belt filter presses, and plate and frame filter presses. They have low energy requirements but cannot sufficiently reduce the water content in many applications (Vaxelaire et al. 1999). Traditional thermal drying methods excel in extracting water. Nevertheless, these methods incur substantial energy consumption for water evaporation, accompanied by significant capital and operational expenses, contingent on the volume of water extracted from the material (Vaxelaire et al. 1999; Mahmoud et al. 2016). Therefore, improving the dehydration capacity of conventional processes is the goal of current research that seeks potential alternatives.

Electroosmotic dewatering is a potential method for extracting water from porous materials by applying a low electric current, which induces electroosmosis phenomena (Vane and Zang 1997). Colloidal particles in sediments/sludge usually have a negative surface charge. They are, therefore, surrounded by a layer with a higher density of positive charges, a phenomenon known as an electric double layer. When an electric field is applied, these negatively charged particles move toward the electrode with the opposite charge. Water from pores and interstitials, along with cations, is driven towards the electrode with the negative charge (Anderson and Idol 1985; Coelho et al. 1996; Weber and Stahl 2002, 2003). Dehydration ensues when water is depleted from the negative electrode (cathode) without replenishment into the positive electrode (anode), generating negative pore water pressure. Electroosmotic dewatering offers several advantages, such as low-energy consumption and no filter media clogging, a common issue in filtration (Yoshida 1993; Visigalli et al. 2017). Therefore, researchers have extensively studied this technique in recent years. Most of the electro-dehydration technologies developed so far use pressurized electro-dehydration. This process applies an electric field, often combined with vacuum filtration or mechanical pressure (Citeau et al. 2011; Visigalli et al. 2017; Wu et al. 2019; Sha et al. 2020; Cao et al. 2021). The pressurized electro-dehydration process is especially suitable for reducing the moisture content of materials that conventional methods have not treated adequately (Bergins et al. 1999; Iwata and Jami 2010; Yang et al. 2018). A wide application area is colloidal systems where strong surface effects complicate dehydration. Due to the significant water–solid bonding forces and potent electrostatic interactions among minuscule particles, achieving mechanical dehydration at ambient temperatures necessitates the application of substantial mechanical forces. Moreover, these combined technologies offer versatile solutions across various applications, including treating fine particle sediments, colloidal sludge, vegetable juice extraction, vegetable waste, and the challenging filtration of activated sewage sludge. Although molecular interactions play a much smaller role, they still dominate the kinetics of water reduction.

Various researchers have extensively studied electroosmotic dewatering, and these works mainly focused on the effects of process parameters, regulation, and process variables, including process design and operational improvements. Some publications also explored how electro-physicochemical properties affect their electrical behavior in terms of electrical dewatering capacity during the pressurized electro-dehydration process (Visigalli et al. 2017). However, due to the complexity of the pressurized electro-dehydration process, it is necessary to study the performance and mechanism of electroosmotic dewatering to better couple it with mechanical or other dehydration methods. Moreover, discussions about changes in sediment characteristics and possible valorization of dewatered sediments were also limited. Applying an electric field could cause the movement of negatively charged organic matter (e.g., fatty acids, humus) from cathode to anode in sediments/sludge, via electrophoresis (Chen et al. 2011; Xiao et al. 2017). Heavy metals and other elements like P in their various forms: in abiotic forms (e.g., soluble, adsorbed, exchangeable, precipitated, and residues) or in biological forms (e.g., extracellular and intracellular species) could also possibly be released and moved under the reaction of an electric field (Kim et al. 2002; Tuan and Sillanpää 2010b, a; Pham-Anh et al. 2012; Tuan et al. 2012). The presence of toxic metals often poses constraints on the agricultural utilization of sediments, making any reduction in heavy metal levels beneficial for both public health and environmental considerations. Changes in the distribution of P and other sediment characteristics influenced by electrochemical and physical reactions produced by the application of electric fields will also affect the utilization of dewatered sediments.

In this study, lake sediments were electroosmotic dewatered without pressure. The changes in moisture distribution were estimated by fitting a step function to the drying curve using non-linear least-squares minimization to investigate the effect of an electric field on sediments’ dewaterability. The main aims are to explore experimentally (1) the performance and mechanisms of electroosmotic dewatering and (2) the overall assessment of characteristics changes of treated sediments and energy consumption to propose potential lake restoration strategies.

## Materials and methods

### Source and preparation of lake sediments samples

The dredged lake sediments sample was obtained from Bagsværd Lake, Copenhagen, Denmark. Large debris, such as leaves and branches, were removed from the sediment by hand, where after the sediment was stored at 5℃ before use.

### Laboratory-scale electroosmotic setup

**Fig. 1:**
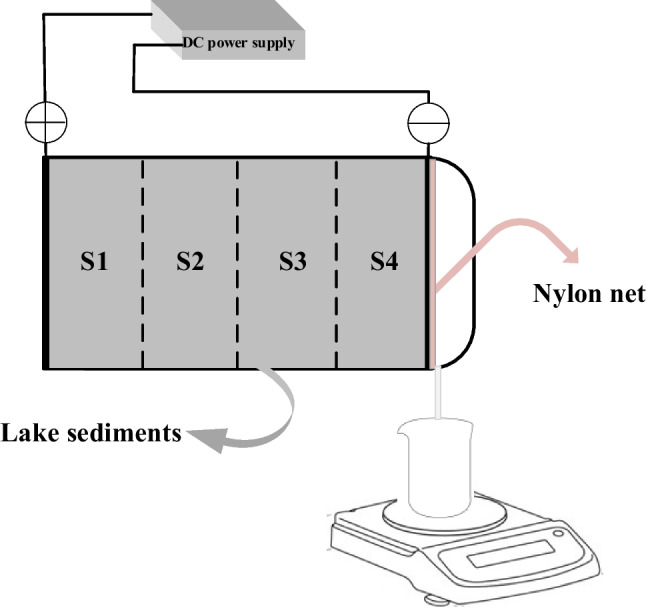
Diagram of the electroosmotic dewatering setup

One liter of sediments was filtrated with gravity before being subjected to the electroosmotic dewatering setup. The electroosmotic dewatering apparatus is a 10-cm long Plexiglas cylinder with a 8-cm internal diameter, as shown in Fig. 1. The sediment samples were applied in sequential layers to enhance homogeneity within the experimental setup, mitigate potential blockages, and ensure uninterrupted drainage. The entire sample is divided into four sections, from near the anode to near the cathode as S1–S4. A constant DC was applied (by a Hewlett Packard E3612A) through two mesh-shaped dimensionally stable electrodes of titanium coated with mixed metal oxides (MMO) to prevent corrosion. The experimental layout is shown in Table 1. A water mass balance was used to monitor the mass of filtrated moisture collected in a beaker. Digital multimeters were used for checking and monitoring voltage and current measurements in electroosmotic dewatering systems. Electrically insulated thermocouples were used to test temperature changes inside the setups in different sections during electroosmotic dewatering, and the computer recorded the experimental data every 60 s.

**Table 1:** Experimental conditions of electroosmotic dewatering

Experiment	A0	B0	C0	A1	B1
Sediments	1-L raw sediments sieved with gravity				
Current	20	40	60	20	40
Duration (hours)	6	6	6	24	24

### Analytical methods

Elements concentration changes of lake sediments before and after experiments were analyzed by ICP-OES before being digested through the US EPA 3015A method. During the digestion, a 10-mL concentrated HNO_3_ was mixed with a 0.5-g dry sediment sample and then heated at 175℃ for 20 min. The pH of the sediments was measured before and after the experiments using both distilled water and dry sediment samples, maintaining a liquid-to-solid ratio (L/S) of 2.5. The measurements were conducted with a radiometer analytical pH electrode. Similarly, the redox potential and conductivity of the sediments were assessed using the same protocol and analyzed with a radiometer analytical ORP electrode. The loss of ignition was tested via placing 2.5 g of dry sediments in the furnace for 1 h at 550 °C. A rapid moisture analyzer (HC103, METTLER TOLEDO, Switzerland) was used to measure the different moisture fractions in the lake sediments by thermal drying at 40 °C (Lee and Hsu 1995). About 5 g of each sediment sample was spread evenly on the balance dish, and the mass change was recorded every 60 s until it reached a constant value. The sample was then heated at 105 °C for 12 h to obtain the final dry mass. The transition points between free, interstitial, surface, and bound moisture were identified by the slope of the drying curves. A step function was fitted to the curves using non-linear least-squares minimization to estimate the moisture content more accurately.

### Calculations

The total energy consumed by the DC power supply to provide a constant current across the setup and to create the electric field was calculated as Eq. (1)

1 $$E=\underset{0}{\overset{t}{\int }}{\text{UId}}_{\text{t}}$$

where *E* represents the energy consumption by the DC power supply (Wh); *t* represents the treatment duration (hours); *I* is the current intensity (A); *U* means the voltage (V).

## Results and discussions

### Characteristics of the sediments

Table 2 shows the main heavy metal content of Cr, Cu, Pb, and Zn, the valuable P content, the main elemental content of Ca and S, the water content, the initial pH values, the conductivity, and the redox potential in raw sediments. The loss of ignition is 47%.

**Table 2:** Characteristics of the sediment sample

Water content (wt.%)	pH values	Conductivity (µS/cm)	Redox potential (mV)
93 ± 1	6.5 ± 0.5	560 ± 50	250 ± 10
Al (mg/kg)	Ca (mg/kg)	Cr (mg/kg)	Cu (mg/kg)
4600 ± 200	17500 ± 500	70 ± 5	100 ± 10
Fe (mg/kg)	K (mg/kg)	Na (mg/kg)	P (mg/kg)
11500 ± 500	1000 ± 70	260 ± 15	800 ± 20
Pb (mg/kg)	Mg (mg/kg)	S (mg/kg)	Zn (mg/kg)
40 ± 5	2150 ± 100	11,000 ± 600	200 ± 20

### Sediments’ electroosmotic dewatering performance

**Fig. 2:**
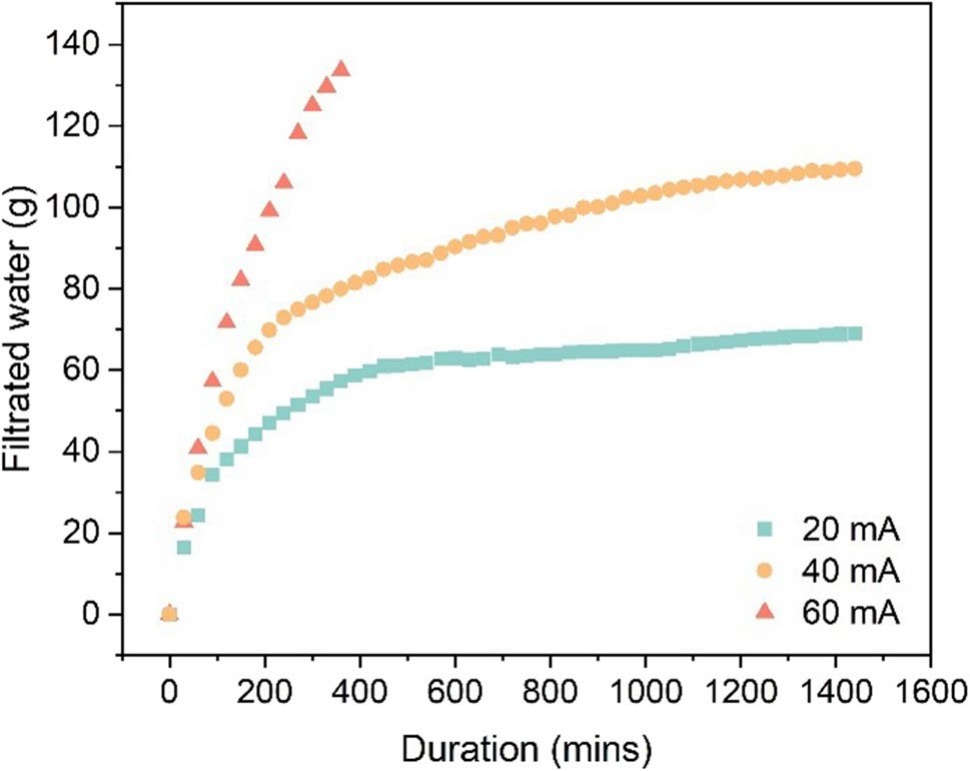
Evolution of filtrate volume with different currents and duration

**Fig. 3:**
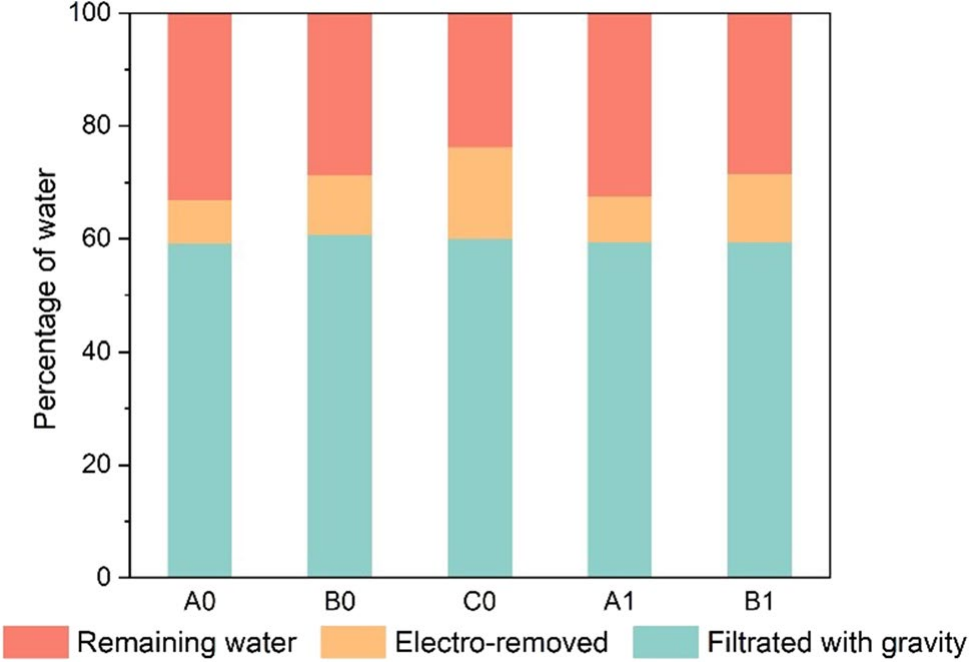
Percentage of removed water and the remaining water

The current density applied during electroosmotic dewatering affects both the final solid dryness and the dewatering kinetics. Figure 2 shows a continuous effluent flow for all the tests during the treatment. The effluent flow and time relationship reveal three distinct phases of electroosmotic dewatering: the fast drain phase, which depends on the electric field strength, the transition phase, and the termination phase. The effluent rate is constant at the initial of filter cake formation, explained by the Helmholtz–Smoluchowski theory (Mahmoud et al. 2010). However, the effluent rate decreases as the dry cake develops with dewatering. After 6 h, more effluent is collected with a higher current. Beyond this duration, there is no significant increase in effluent, especially at a lower current. As shown in Fig. 3, the percentage of water removal and the remaining water in dewatered sediments at the end indicate that gravity filtration can eliminate about 60% of the moisture. Applying an electric field (20 mA, 40 mA, and 60 mA) can further reduce the moisture content by 7–20%, significantly decreasing the mass for transportation or storage. However, 20–35% of the moisture remains in the sediments after the experiments.

**Fig. 4:**
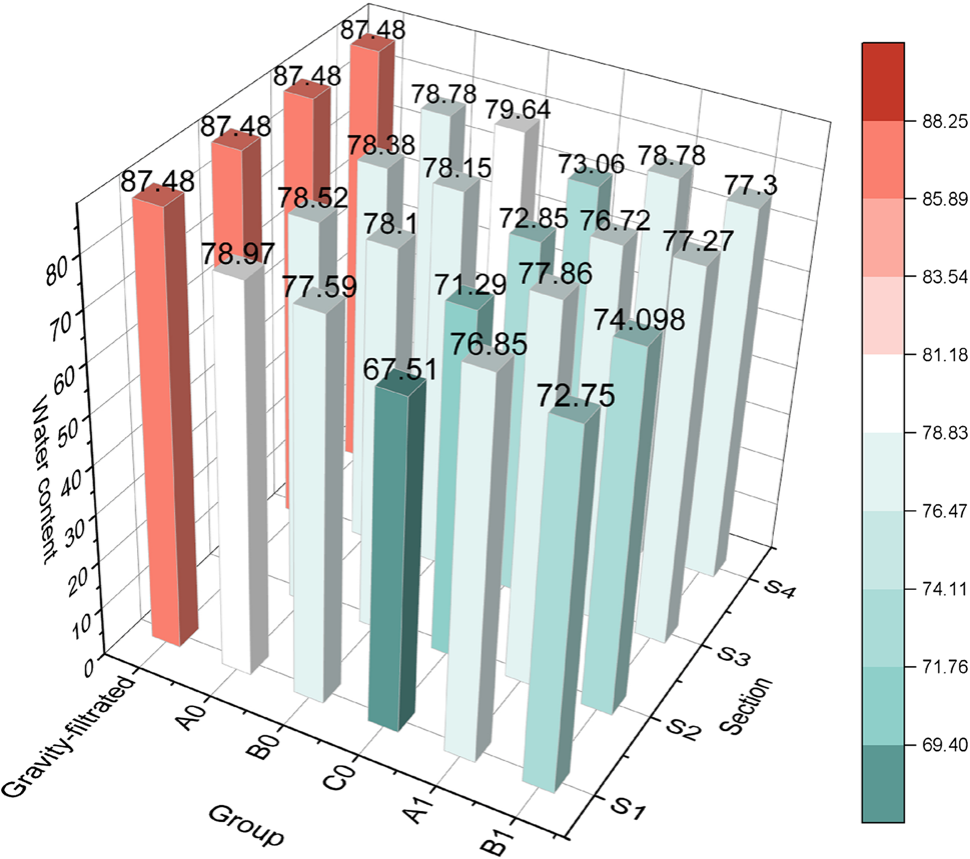
Changes in water content

Figure 4 shows that the water content of the sediment samples was significantly reduced by electroosmotic dewatering after gravity filtration. The initial water content of about 93% was lowered to around 85% by gravity filtration and then further reduced to about 65 to 80% by electroosmotic dewatering. The effect of electroosmotic dewatering on the water content depended on the applied current and duration. After 6 h of electroosmotic dewatering with a current of 20 mA, the water content of the samples in each section was similar. It did not change significantly with a further increase in duration. However, when a current of 60 mA was applied, or 40 mA was applied for a longer duration, the water content of the samples varied from anode to cathode, showing a stepwise pattern. The samples near the anode had slightly lower water content than those near the cathode, possibly due to the electroosmotic flow from the anode to the cathode. Moreover, the higher current increased the system voltage, resulting in more ohmic heating and changes in pH and redox potential due to electrode reactions. These factors affected the sediment characteristics and enhanced the release and removal of water. When using a current of 60 mA, voltage fluctuations were observed after a few hours, possibly attributed to phenomena such as crack formation at the anode and bubble formation at the electrodes due to water electrolysis. These phenomena could reduce the contact between the sediments and the electrodes, increase resistance, and affect the stability of the dewatering system voltage (Mahmoud et al. 2010).

### Changes in moisture distributions

**Fig. 5:**
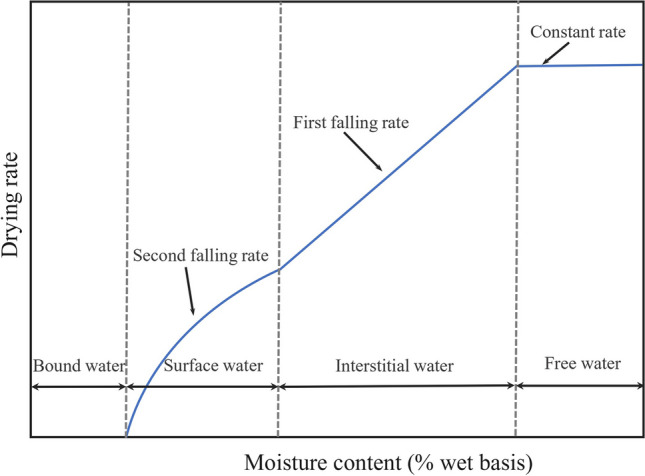
Typical drying curve

The water within the sediments behaves differently with regard to factors such as enthalpy, entropy, vapor pressure, viscosity, and density due to the presence of solids and differs in the difficulty of removal (Vaxelaire and Cézac 2004). The thermal drying test was first proposed by Tang and Vesilind to investigate the moisture distributions based on the analysis of the drying curve (Deng et al. 2011). The drying curve shows the evolution of evaporation flux versus the average moisture content and classically presents four phases (Fig. 5). As the moisture content decreases, the classical drying curve shows a constant rate period, a first falling rate period, a second falling rate period, and finally, an equilibrium period. The different periods present the bond type between the solid particles (or material structure) and the water (Tsang and Vesilind 1990; Robinson and Knocke 1992). The constant rate period shows the free (unbound) water evaporation at the particle surfaces. Then, the drying boundary progresses into the hygroscopic materials, and the first falling rate period appears. During the first falling rate period, the evaporated water may mainly be mechanically bonded with solid particles (e.g., capillary trapped inside interstitial spaces of organisms and flocs) and is classified as interstitial water, the increase of mass and heat transfer resistance resulting in evaporation flux decrease. The second falling rate period appears owing to the evaporation of more hardly bounded water (surface water). That water may be physically adsorbed or adhesive onto the solid particle surface. During the equilibrium period, the remaining bond water is chemically bonded (e.g., intracellular or internal water) by powerful linkage and removed at 105℃ for 12 h. Accurately defining the critical points referring to transitions between the constant rate and the first falling rate period, as well as the first falling rate and second falling rate period, is essential for distinguishing different moisture content. Thus, the step function was defined to describe the development of the drying curve, and non-linear least-squares minimization and curve fitting were applied to determine the critical points more accurately.

**Fig. 6:**
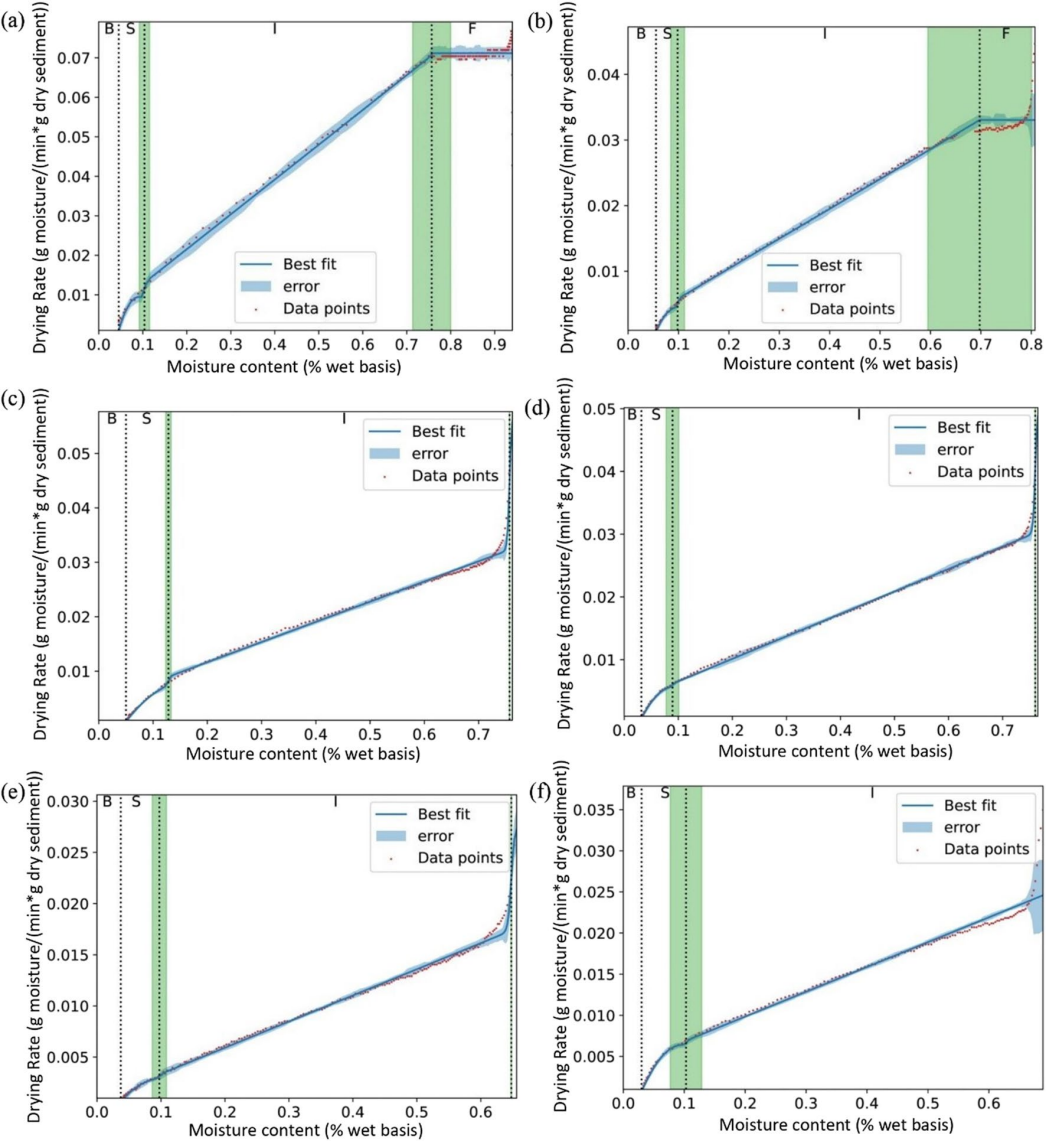
Drying curve of raw sediments (**a**), sediments after filtration with gravity (**b**), A0-S1 (**c**), A0-S4 (**d**), C0-S1 (**e**), and C0-S4 (**f**). B, bond water; S, surface water; I, interstitial water; F, free water

The drying curves of raw sediments, sediments after filtration with gravity, A0-S1, A0-S4, C0-S1, and C0-S4 were shown in Fig. 6. From Fig. 6a, the results show that four periods are present clearly when drying the raw sample, indicating all four types of moisture existed in the raw sediments. The critical point between free and interstitial water was observed around 70–80% moisture content basis. This shows that free water in raw sediments only accounts for small proportions, which may explain why reducing the water content to below 70% with electroosmosis was difficult, whether using a higher current or a longer duration (Fig. 4). Interstitial water was found around 10–80% moisture content basis and accounts for the major proportion in raw sediments. The surface water and bond water were observed around 5–10% and 0–5% moisture content basis, respectively. After filtration with gravity, parts of free water were removed, and other moisture content was not influenced significantly. In general, the drying curve of sediments after electroosmotic dewatering presents similar tendencies as raw and gravity-filtrated sediments. However, after electroosmotic dewatering, most free water was removed, as the constant rate period of the drying curve could not be detected. Even some interstitial water was removed with the application of high current (Fig. 6e). However, the surface water and bound water might not be affected significantly. Similar phenomena were observed in the study of Tsang and Vesilind when investigating moisture redistributions of sludge after being drained, filtered, and centrifuged (Tsang and Vesilind 1990).

### Influence of electrochemical reaction on dewaterability

**Fig. 7:**
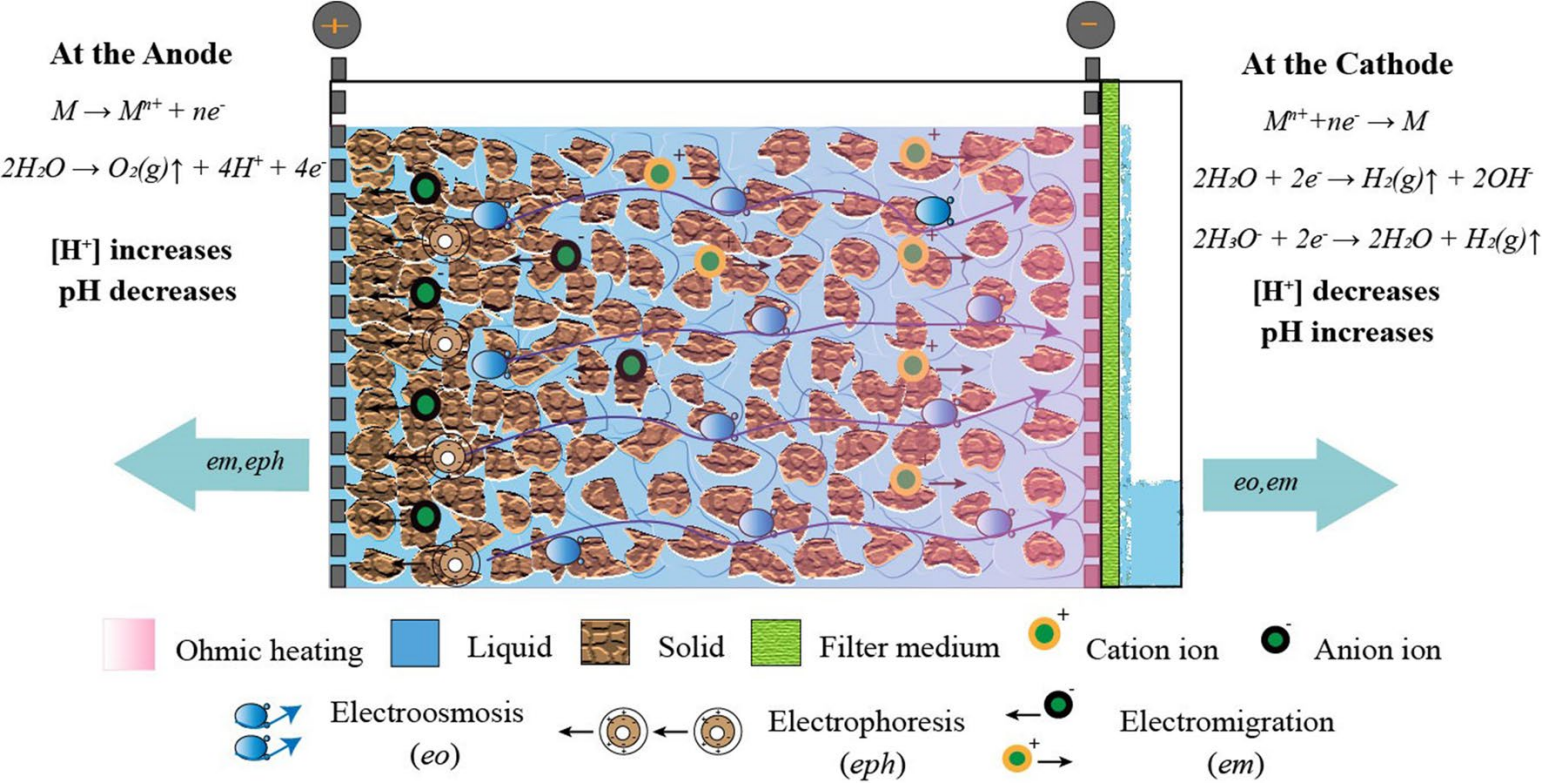
Schematic representation of the electroosmotic dewatering with different mechanisms of liquid and solids occurring when lake sediments is placed in an electrical field

The moisture distribution analysis reveals that even a lower current can significantly remove the free water in the raw sediments after 6 h, as the constant rate period of the drying curve could not be detected in all the tests for the electroosmotic treated samples (Fig. 6). Consequently, the differences in the final water content can be attributed to the release of interstitial water. Figure 7 shows the main mechanisms of liquid and solids occurring during electroosmotic dewatering. The application of current induced the movement of moisture within sediments (electroosmosis). Electrochemical reactions induced by the electric current impact the characteristics of the sediments, including conductivity and pH. These electrochemical reactions, coupled with other electrokinetic phenomena such as electromigration and electrophoresis, affect sediment properties and moisture status. Varying the current intensity in the electroosmotic dewatering system results in changes in the strength of the electrochemical reaction. These discrepancies influence the duration and timing of the rapid drain phase, transition phase, and termination phase during electroosmotic dewatering (Fig. 2). With a higher current, the fast drain and transition phases are prolonged, and the presence of transition and termination phases are delayed, leading to differences in the final water content.

The temperature development also shows significant differences under different currents, which might affect the release of interstitial water in different experimental groups. Ohmic heating could increase and change the temperature inside the sediments during electroosmotic dewatering (Navab Daneshmand et al. 2012). Ohmic or Joule heating is the heat generated due to the electrical current flowing through sediments and the increased resistance (Navab-Daneshmand et al. 2015). According to Joule’s law, the ohmic heating is expressed as Eq. (2), and the *R*_cell_ can be calculated as Eq. (3)

2 $$Q= {I}^{2}{R}_{\text{cell}}$$

3 $${R}_{\text{cell} }= {U}_{\text{Cake} \text{sediments} \text{bed}}/I$$

where *Q* is the ohmic heating (J); ${R}_{\text{cell}}$ presents electrical resistance (ohm); *I* is the electric current (A); ${U}_{\text{Cake sediments bed}}$ is the ohmic drop in the sediments cake bed.

**Fig. 8:**
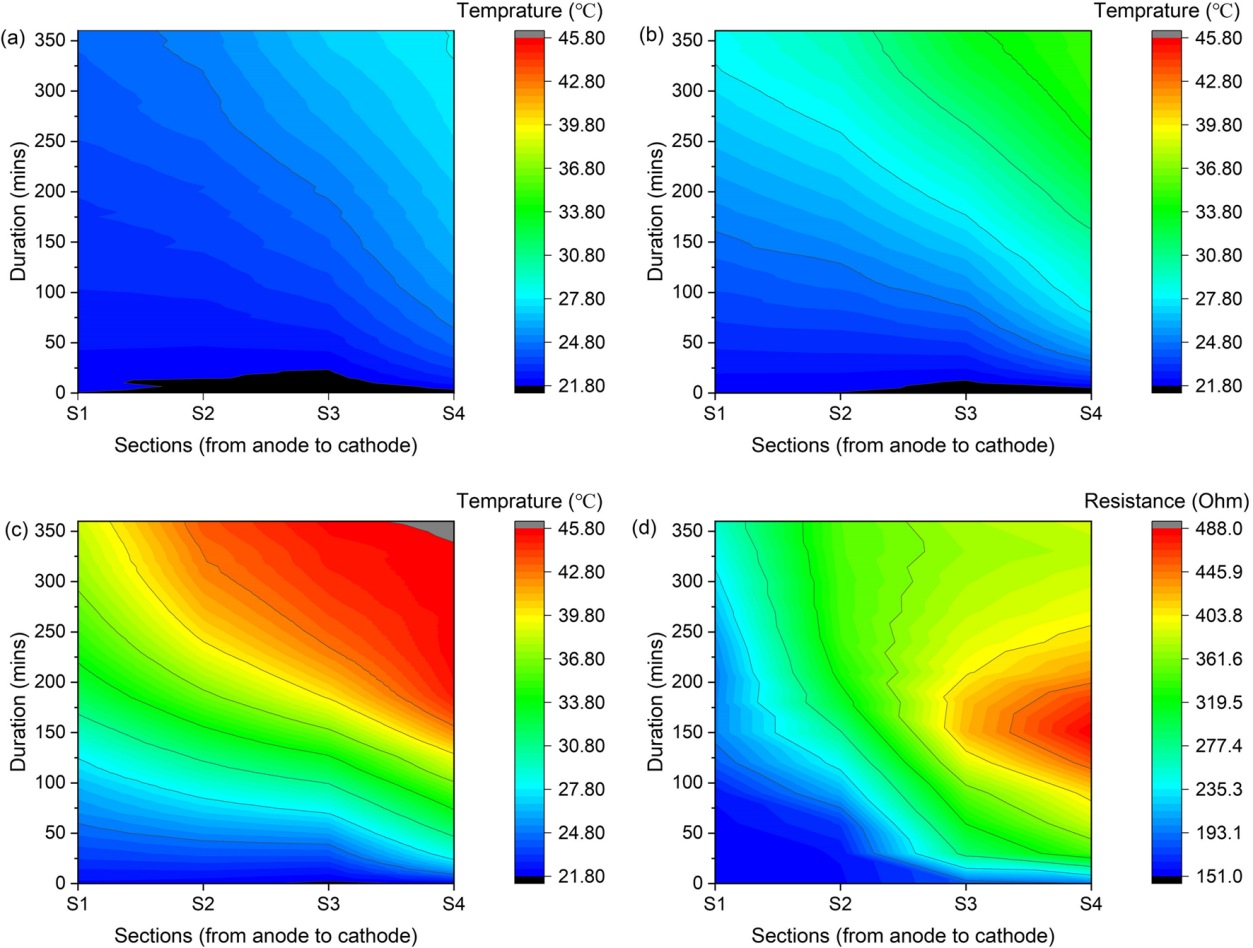
Development of temperature in group A0 (**a**), B0 (**b**), and C0 (**c**), and resistance changes of group C0 (**d**)

The ohmic heating plays a substantive role during electroosmotic dewatering. It internally heats the sediments and raises the temperature, which will decrease the viscosity of the liquid, potentially expelling moisture from the solid capillaries (Clayton et al. 2006; Mahmoud et al. 2011) and accelerating electroosmotic flow. The development of temperature varies under different currents, as shown in Fig. 8a, b, and c. Generally, the temperature rise is more pronounced for incremental increases in applied current. The average temperature of sediments in the setup was in the range of 20–28℃ (A0), 20–36℃ (B0), and 20–47℃ (C0). Calculating the change in dynamic water viscosity corresponding to these temperature ranges and normalizing with its value at 20 °C (${\eta }_{(T)}/{\eta }_{(20^\circ{\rm C} )}$). The corresponding decrease in viscosity is 5–15% in the case of A0, 15–30% in the case of B0, and 30–45% in the case of C0. Therefore, the contribution of ohmic heating in reducing viscosity fundamentally accelerates the dehydration kinetics, facilitating the removal of typically stubborn remaining water. In this way, ohmic heating synergistically complements the electroosmotic flow, leading to an enhanced dewatering process and improved kinetics, consistent with the observations made by Mahmoud et al. (Mahmoud et al. 2016).

Besides, the temperature rises faster in the area near the cathode than in the area near the anode, which may be related to the resistance changes. Figure 8d shows the development of resistance in C0. Generally, the resistance increased with the continuous reduction of moisture content and formation of sediments cake. However, it rises faster in the area near the cathode than in the area near the anode. Wyllie et al. developed a model decades ago based on the “porous plug” model, which considers three different paths for electrical current flow through the bed (Sauer et al. 1955; Mahmoud et al. 2006). These include (1) alternating layers of interstitial solution and particles, (2) particles in contact with each other, and (3) in the liquid phase channel. Thus, according to the model, the flow of electric current through porous media is determined by the conductivity of the solid (dispersed phase) and liquid phase (continuous phase). The relationship between the conductivity of the medium (*k*) and ohmic resistance (*R*) can be described as Eq. (4):

4 $$k= \frac{1}{R} \frac{L}{S}$$

where *L* is the electrode gap and *S* is the electrode area. For all the experimental groups, *L* and *S* were the same. Thus, resistance is inversely proportional to conductivity. During the electroosmotic dewatering process, the electrochemical reaction affects the conductivity of liquids and solids. The possible cathode and anode reactions are shown in Fig. 7. Therefore, the dissolution of substances near the anode is beneficial to the maintenance of liquid and solid conductivity. Precipitation of species near the cathode exacerbates the decrease in conductivity and the increase in resistance. Moreover, it is noticed that the resistance of the sample near the cathode area also showed a sudden short-term growth. This phenomenon may be related to the generation of gas evolution that causes voids in the bed, creating an electrically insulating layer and significantly increasing the electrical resistance of the system. This can cause an increase in energy consumption and affect system stability. During electroosmotic dewatering, the negatively charged sediment particles move toward the anode under electrophoresis, increasing the compactness and strength of the sediments near the anode. Therefore, forming temporary holes near the cathode might be more conducive.

In summary, electroosmotic dewatering leverages electroosmotic phenomena, coupled with ohmic heating, electrochemical reactions, and other electrokinetic phenomena, to effectively remove water from the lake sediments, thereby influencing their dewatering and activation processes. According to the changes in temperature, it is found that lower current leads to slower and more uniform temperature changes and water distribution in each region of the sample. A higher current causes faster and more uneven temperature changes and does not result in lower water content or more interstitial water release near the hotter cathode. This indicates that excessive ohmic heating may not be effective for moisture removal. Therefore, proper design to control the ohmic heating in each region within a specific range may reduce the electric energy loss and improve the energy efficiency of electroosmotic dewatering.

### Cost estimates and overall assessment of the practical application

**Fig. 9:**
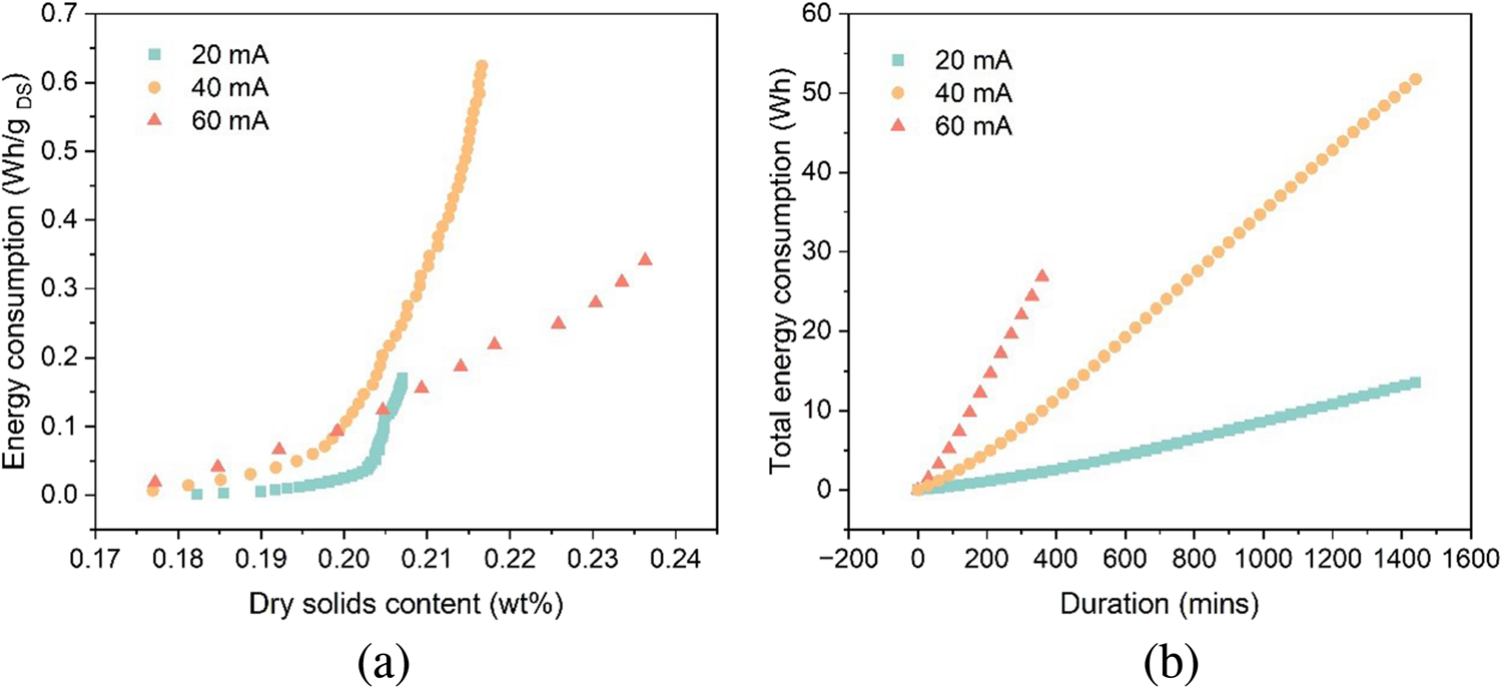
Energy consumption per unit of dry basis (**a**) and the total energy consumption (**b**)

Electrochemical technology has been widely used for environmental management by many researchers. It can offer effective solutions for moisture removal, environmental protection, materials recycling, and clean synthesis by applying and improving existing methods or developing and implementing new ones (Walsh 2001; Janssen and Koene 2002; Reddy et al. 2006; Anjum et al. 2017). However, some challenges remain for electrochemical-assisted technologies, such as electroosmotic dewatering, to reach a high technology readiness level and become marketable processes (Lacasa et al. 2019). One of these challenges is to improve economic feasibility. Hence, the energy consumption of electroosmotic dewatering was analyzed to propose possible strategies for reducing the operational costs of this technique. After gravity filtration and electroosmotic dewatering, 1 L of sediments can reduce water content to 65–75%, comparable to low pressurized filtration (e.g., belt filter), with a low-energy consumption (Chen et al. 2002; Huttunen et al. 2017). In general, the total energy consumption increases with time, as shown in Fig. 9b. Energy consumption rates of 0.042 kWh/kg _water removed_, 0.119 kWh/kg _water removed_, 0.201 kWh/kg _water removed_, 0.197 kWh/kg _water removed_, and 0.473 kWh/kg _water removed_ were observed during the application of different current densities and durations: 20 mA for 6 h (A0), 40 mA for 6 h (B0), 60 mA for 6 h (C0), 20 mA for 24 h (A1), and 40 mA for 24 h (B1), respectively. Of these conditions, enhanced moisture removal was observed with the application of 40 mA for 24 h and 60 mA for 6 h. The energy consumption associated with the 6 h application of 60 mA was considerably lower than that of applying 40 mA for 24 h. When considering the theoretical energy requirement for thermal drying, estimated at approximately 0.617–1.2 kWh per kg of evaporated water, it shows that the electroosmotic dewatering technique offers significantly higher energy efficiency (Mahmoud et al. 2011). Moreover, the expense associated with electrodes for electroosmotic dewatering is notably lower compared to equipment required for alternative dewatering methodologies. At the beginning of electroosmotic dewatering, the energy consumption increases slowly, and then the growth rate gradually accelerates. Figure 9a shows that the higher the current used, the faster the growth of energy consumption. It proves that the energy consumption in the electroosmotic dewatering process is not constant but depends on the cake’s dryness. The higher the cake’s dryness, the higher the energy consumption. This is attributed to the increased difficulty of removing the remaining water due to the enhanced binding strength. Moreover, the drying cake and the gas generated near the electrodes may impair the electrical contact between the cake and the electrode, resulting in increased contact resistance and reduced electric field inside the cake, which consequently lowers the driving force. Precipitates generated near the cathodes may also cause an increase in resistance, thus lowering the driving force. Therefore, when electroosmotic dewatering is applied or combined with other techniques, a reasonable design should consider the better contact between the cake and the electrodes, the sediments particles, and the efficient gas release generated by electrolysis. The pretreatment of sediments (e.g., using flocculants) can also help reduce energy consumption by weakening the bond between the remaining moisture and the sediments particles. Adding an acidic buffer near the cathode may reduce the energy loss from higher ohmic heating near the cathode by alleviating precipitate generation. Besides, it is found that using a higher current for a shorter period reduces the water content of the sediments more effectively while consuming less power than using a lower current for a more extended period. Thus, the requirement of the final water content by electroosmotic dewatering should also be fully considered. For example, the duration should be controlled to end at the fast drain phase and before the transition phase appears (Fig. 2) to maintain high-energy efficiency. On this basis, if the duration is further increased, some more water can be removed, but it may significantly increase energy consumption. After the transition phase ends and enters the termination phase, continuing to apply electric current will not significantly affect water removal. Still, longer electrochemical reactions may increase the change of sediment characteristics and affect suitable reuse methods for treated sediments.

**Fig. 10:**
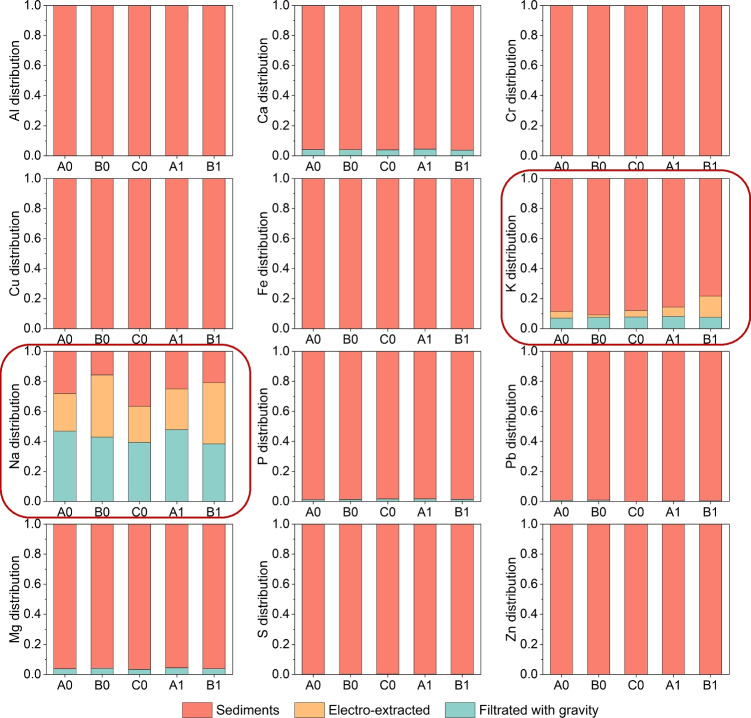
The normalized distribution of main elements at the end of the electroosmotic dewatering

**Fig. 11:**
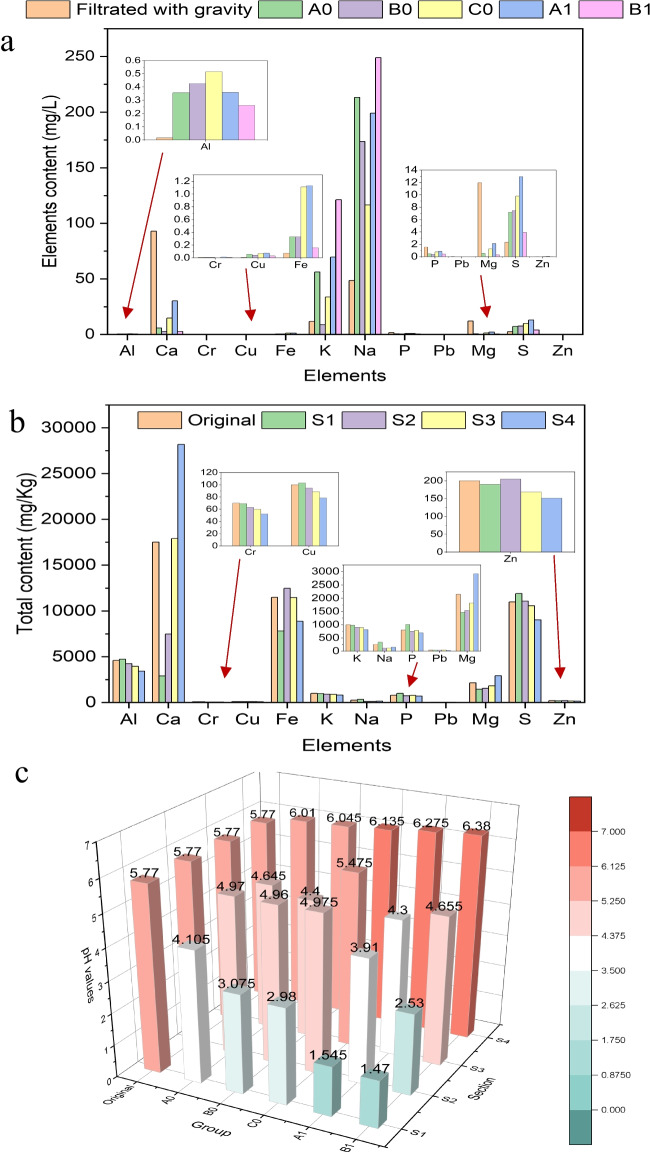
Elements concentration in the water filtrated with gravity and electroosmotic removed water (**a**); the total content of different elements from raw sediments and sediments electroosmotic dewatered with 40 mA current after 24 h (**b**); pH changes after electroosmotic dewatering (**c**)

Electroosmotic dewatering is a technique that uses an external electric field to extract water from semi-solid materials placed between two electrodes, forming an electrochemical cell. Besides electroosmosis, other electrokinetic phenomena involved in this process, such as electrode reactions, electrophoresis, and electromigration, can affect the distribution of elements in the sediments and thus influence the reuse value of the treated sediments. The effects of electroosmotic dewatering on the redistribution of elements such as heavy metals and P were investigated. The results showed that most of these elements were not effectively removed by electroosmotic dewatering (Fig. 10). But around 1–15% of K and 20 – 45% of Na were extracted. Figure 11a shows that the main components of the removed water were K, Na, and trace amounts of Ca by electroosmotic dewatering. The water contained negligible amounts of toxic heavy metals or essential elements of P. Therefore, the removed water could be used as a Na and K supplement or disposed of directly. When a higher current is used for a shorter time, more liquid is extracted in a shorter time, and the concentration of elements contained in the extracted solution is smaller. This facilitates disposing of the extracted solution, such as directly returning it to the lake. The total content of some elements and pH values also changed after treatment. Generally, longer durations result in more noticeable changes in elemental composition and the pH values of the sediments. Figure 11b shows that after applying 40 mA for 24 h, Ca, Fe, and Mg concentrations decreased near the anode and increased near the cathode, while P concentration showed the opposite trend. The pH values of the treated sediments changed due to water electrolysis, with lower pH near the anode and higher pH near the cathode (Fig. 11c). These variations in elemental distributions and pH suggest different potential applications for the treated materials from different sections. For instance, the treated sediments near the anode with higher P content, lower Ca, Fe, and Mg content, and lower pH could be suitable for P extraction and recycling by methods such as electrodialysis. The extraction of P elements is usually more accessible at lower pH levels. The content of the Ca element will affect the buffer capacity of sediments. Mieczysław finds that Ca can form complexes with organic matter and clay minerals by replacing other cations on the sorption complex and that these complexes can bond H^+^ ions and neutralize acids (Nehls and Wessolek 2011). The presence of Fe and Mg elements may affect the P mineralogy and extraction efficiency (Ohtake and Tsuneda 2018). The treated sediments near the cathode with higher Ca content and pH could be suitable for producing building materials such as bricks and cement (Samara et al. 2009).

## Conclusions and perspective

The study explored the performance and potential of electroosmotic dewatering for dewatering and valorizing phosphorus-rich lake sediments. Based on the analysis of the drying curve, the non-linear least-squares minimization and curve fitting were applied to determine the different moisture content more accurately. The conclusions may be summarized as follows:

- Applying an electric field (20 mA, 40 mA, and 60 mA) to 1-L sediments after gravity filtration can further reduce the mass of sediments (7–20%) and lower transportation or storage costs.
- Moisture distribution analysis shows all four types of free, interstitial, surface, and bond water existed in raw sediments. The electroosmotic removed water is mainly free water, and some released interstitial water.
- The fast drain phase, the transition phase, and the termination phase occur during electroosmotic dewatering. The optimal duration of electric current application should be considered to balance water removal and energy consumption.
- A higher current (60 mA) can enhance dewatering efficiency with lower energy consumption (0.201 kWh/kg _water removed_), where ohmic heating may help to decrease viscosity and release the water from the capillary. However, it may result in excess ohmic heating, more precipitating, and gas generation (especially near the cathode), thus decreasing energy efficiency and compromising the system’s stability.
- The current conditions do not significantly extract heavy metals or P from the sediments, which may facilitate the disposal of the removed water. The notable changes in metals and P distribution and pH values imply that treated sediments from different sections of setups could be reused for different purposes such as construction and P extraction.

Therefore, this technology is easy to operate and suitable for the treatment of dredged sediments. Future work should aim to improve the contact between the cake and the electrodes, the sediments particles, and the efficient release of gas. It should also consider pretreatment to weaken the bond between the remaining moisture and the sediment particles, and alleviate the excess ohmic heating to improve the electric energy efficiency for commercial viability.

## Data Availability

The authors declare that the data supporting the findings of this study are available within the paper. Should any raw data files be needed in another format they are available from the corresponding author upon reasonable request.
